# Genome Wide Association Study and Next Generation Sequencing: A Glimmer of Light Toward New Possible Horizons in Frontotemporal Dementia Research

**DOI:** 10.3389/fnins.2019.00506

**Published:** 2019-05-16

**Authors:** Miriam Ciani, Luisa Benussi, Cristian Bonvicini, Roberta Ghidoni

**Affiliations:** ^1^Molecular Markers Laboratory, IRCCS Istituto Centro San Giovanni di Dio Fatebenefratelli, Brescia, Italy; ^2^Department of Molecular and Translational Medicine, University of Brescia, Brescia, Italy

**Keywords:** frontotemporal dementia, genome wide association study, next generation sequencing, genetic mutations, genetic rare variants, genetic common variants

## Abstract

Frontotemporal Dementia (FTD) is a focal neurodegenerative disease, with a strong genetic background, that causes early onset dementia. The present knowledge about the risk loci and causative mutations of FTD mainly derives from genetic linkage analysis, studies of candidate genes, Genome-Wide Association Studies (GWAS) and Next-Generation Sequencing (NGS) applications. In this review, we report recent insights into the genetics of FTD, and, specifically, the results achieved thanks to GWAS and NGS approaches. Linkage studies of large FTD pedigrees have prompted the identification of causal mutations in different genes: mutations in *C9orf72*, *MAPT*, and *GRN* genes explain the large majority of cases with a high family history of the disease. In cases with a less clear inheritance, GWAS and NGS have contributed to further understand the genetic picture of FTD. GWAS identified several common genetic variants with a modest risk effect. Of interest, many of these variants are in genes belonging to the endo-lysosomal pathway, the immune response and neuronal survival. On the opposite, the NGS approach allowed the identification of rare variants with a strong risk effect. These variants were identified in known FTD-associated genes and again in genes involved in the endo-lysosomal pathway and in the immune response. Interestingly, both approaches demonstrated that several genes are associated to multiple neurodegenerative disorders including FTD. Thanks to these complementary approaches, the genetic picture of FTD is becoming more clear and novel key molecular processes are emerging. This will foster opportunities to move toward prevention and therapy for this incurable disease.

## Introduction

Genetic understanding of neurodegenerative diseases has considerably increased over the years, favoring the identification of possible targets for new potential therapies.

In this review, we report the most recent genetic insights regarding Frontotemporal Dementia (FTD), a focal neurodegenerative disease affecting the frontal and temporal lobes of the brain. FTD has a heterogeneous clinical presentation: behavioral abnormalities are prominent in the behavioral variant FTD (bvFTD) (Rascovsky et al., 2011), while language disabilities characterize the Primary Progressive Aphasias (PPAs) (Gorno-Tempini et al., 2011). In addition, the FTD clinical presentation may include movement disorders, such as Progressive Supranuclear Palsy (PSP) and Corticobasal Syndrome (Litvan et al., 1996; Armstrong et al., 2013). Once considered a rare disorder, nowadays FTD is considered a common form of early-onset dementia, with a mean age of presentation under 65 years old (Ratnavalli et al., 2002; Knopman et al., 2004; Coyle-Gilchrist et al., 2016).

Frontotemporal Dementia has a strong genetic background: 30–40% of FTD patients have a positive family history and, even if a clear autosomal dominant inheritance pattern is often difficult to trace, a high family history is present in roughly 15% of FTD patients (Rohrer et al., 2009; Wood et al., 2013; Fostinelli et al., 2018). Linkage studies of large families with dominant inheritance pattern for FTD have led to the identification of causative mutations in different genes. Mutations in the microtubule associated protein tau gene (*MAPT*), located on chromosome 17 and encoding for tau protein, were first identified in 1998 in FTD patients with tau-positive brain inclusions (Hutton et al., 1998; Poorkaj et al., 1998). Genetic research in FTD remained almost silent till 2006, when null mutations in the *GRN* gene, encoding for progranulin, were found in FTD families with a positive linkage on chromosome 17, in the proximity of *MAPT* (Baker et al., 2006; Cruts et al., 2006). So far, all identified pathogenic *GRN* mutations exert a null effect on progranulin protein: therefore, mutations can be easily captured by plasma/serum dosage (Ghidoni et al., 2008, 2012; Finch et al., 2009; Sleegers et al., 2009). In 2011, an additional major genetic determinant was found in families with FTD and Amyotrophic lateral sclerosis (ALS): an intronic expansion of a hexanucleotide repeat in the *C9orf72* gene, located on chromosome 9, where previous linkage studies had had identified an FTD/ALS locus (DeJesus-Hernandez et al., 2011; Renton et al., 2011). *MAPT*, *GRN*, and *C9orf72* mutations, detected in up to 74% of patients with high family history, represent the most frequent genetic cause of FTD; nowadays 54 and 79 mutations have been described for *MAPT* and *GRN*, respectively.^1^,^2^ Of note, a compound heterozygosity of two *MAPT* mutations (transmitted by the unaffected parents) was found in a sporadic case, thus highlighting that mutations can be found also in sporadic cases (Anfossi et al., 2011). However, considering FTD patients with lower family history and apparently sporadic cases, mutations in these genes are found in roughly 15% of cases (Wood et al., 2013; Fostinelli et al., 2018). Additionally, rare FTD-causing mutations have been found in: the Valosin gene (*VCP*), that was first reported to be mutated in families with hereditary inclusion body myopathy with Paget disease of the bone and FTD; the Charged multivesicular body protein 2B (*CHMP2B*), with only one proven pathogenic mutation described in 2005 and segregating with FTD; the TAR DNA binding protein (*TARDP*) and the Fused in Sarcoma (*FUS*) genes, two well established ALS-associated genes, playing a minor role also in FTD (Pottier et al., 2015). Together, mutations in these genes account for a large proportion of FTD pedigrees with a high family history of the disease, but only a minority of apparently sporadic patients or patients with a less clear family history.

Thus, deciphering the “missing heritability” of the remaining FTD cases represents one of the main challenges in FTD research. In this direction, Genome-Wide Association Studies (GWAS) and Next-Generation Sequencing (NGS) technologies represent a great potential.

## Genome Wide Association Studies and the “Common-Variants Theory”

For many years, the candidate-gene hypothesis and genetic linkage studies have been the predominant approaches to guide the discovery of FTD-associated genes (Loy et al., 2014). However, these approaches were not enough to fill the gap regarding the missing heritability of most cases. Thus, the candidate-gene studies have been completed with hypothesis-free approaches like GWAS studies, which are based on the analysis of common variants widely distributed in the genome, with a modest effect (Yang et al., 2010; Weiner et al., 2017). Here, the “common-variants theory,” which claims that common disease-causing variants can be identified in every human population that manifest a given disease, became the predominant molecular paradigm. GWAS is based on the use of specific genotyping arrays that interrogate an independent set of variants within the whole genome in related/unrelated individuals, identifying an association between one/more variants and pathological traits (Mishra et al., 2017). This approach has allowed the identification of genetic alterations conferring disease risk revealing that susceptibility factors can be enriched in genes clumped into disease-relevant pathways, offering new angles for research and therapeutic intervention (Ferrari et al., 2015).

A number of genomic regions that may increase the FTD risk has been identified (Table 1).

**TABLE 1:** Genome-Wide Association Studies (GWAS) mediated identification of potential pathways contributing to FTD.

Genes	Pathways	References
		Van Deerlin et al., 2010;
		Finch et al., 2011;
		Busch et al., 2016;
*TMEM106B*	Lysosomal function	Nicholson and Rademakers, 2016;
		Gallagher et al., 2017;
		Rhinn and Abeliovich, 2017;
		Pottier et al.,2018
*HLA*	Immune response	Ferrari et al., 2014; Broce et al.,2018
*RAB38, CTSC*	Vesicle-trafficking; lysosomal function	Ferrari et al.,2014
*C9orf72, UNC13A*	Neuronal survival	Karch et al.,2018
*HLA, MAPT, APOE*	Intracellular vesicular trafficking; immune response; endo/lysosomal processes	Ferrari et al.,2017
*GFRA2*	Neuron survival and differentiation	Pottier et al.,2018
C6orf10/LOC101929163	Microglial/autophagy pathways	Zhang et al.,2018
*LRRK2, TBKBP1 5PGBD5*	Enzyme function; immune response; sequence-specific genomic rearrangements	Broce et al.,2018
*ELP2*	RNA transcription	Taskesen et al.,2018
*APOE, TOMM40 SERPINA1, ARHGAP35*	Lipid metabolism; metabolic and mitochondrial pathways Stress-signaling pathway Stress-signaling pathway	Mishra et al.,2017
*RFNG, AATK*	Neuronal genesis and differentiation; axon outgrowth	Ferrari et al.,2015
*ABCA7, DYSF, PAXIP1*	Lipid metabolism; immune response; membrane regeneration and repair: genome stability; Cell survival	Chen et al.,2015

The importance of the transmembrane protein 106B (*TMEM106B*) gene, identified by the first GWAS on FTD patients (Van Deerlin et al., 2010), has been confirmed in different studies (Busch et al., 2016; Nicholson and Rademakers, 2016; Gallagher et al., 2017; Rhinn and Abeliovich, 2017). In Van Deerlin et al. (2010), the first team recognized *TMEM106B* as genetic risk factor in patients with a specific neuropathology. However, when the authors tried to validate this result in a more heterogeneous group of probable FTD cases, lacking post-mortem confirmation, the association of *TMEM106B* with FTD was lost. Interestingly, van Blitterswijk et al. (2014) identified *TMEM106B* variants which appeared to alter the *C9orf72* phenotype and cause later disease onset. Similarly, specific variants in this gene influence *GRN*-associated FTD risk, reducing the disease penetrance in *GRN* mutation carriers (Finch et al., 2011). Latecomer, the FTD risk linked to *TMEM106B* variants seems to be associated to lysosomal dysfunctions, being *TMEM106B* a lysosomal protein (Nicholson and Rademakers, 2016; Klein et al., 2017). Recently, Pottier replicated the previously reported *TMEM106B* association and identified a novel genome-wide significant locus at the GDNF family receptor alpha 2 (*GFRA2*) gene, which encodes for a neurotropic factor with a key role involved in neuron survival and differentiation (Pottier et al., 2018). Moreover, in a recent GWAS study, a link between the C6orf10/LOC101929163 locus and the age of onset in *C9orf72* mutation-carriers was identified, supporting the involvement of autophagy in modulating *C9orf72* disease (Zhang et al., 2018).

In the largest FTD-GWAS so far, the *HLA*, *RAB38*, and Cathepsin C (*CTSC*) genes were recognized as FTD risk loci, suggesting alterations in immune system, lysosomal and autophagic pathways (Ferrari et al., 2014). An immune-related genetic enrichment in FTD was also described in a very exhaustive study in which a systematic investigation of genetic overlap between immune-mediated diseases and the spectrum of FTD-related disorders was performed. In addition, the authors identified novel susceptibility loci within the Leucine rich repeat kinase 2 (*LRRK2*), the TBK1 binding protein 1 (*TBKBP1*), and the PiggyBac transposable element derived (*5PGBD5*) genes, involved in cell survival, immunity processes and genomic rearrangements, respectively (Broce et al., 2018). Interestingly, GWAS was also used to evaluate shared pathobiology between neurodegenerative disorders. Recently, Karch et al. (2018) highlighted a genetic overlap between FTD and ALS, identifying shared common variants near *C9orf72* and the Unc-13 homolog A (*UNC13A*) genes, linked to neuronal vitality. Additionally, the Major histocompatibility complex (*HLA*), the *MAPT*, and the Apolipoprotein E (*APOE*) regions were associated to FTD, Alzheimer’s disease (AD) and Parkinson’s Disease (PD) risk, supporting a genetic pleiotropy in these neurodegenerative diseases (Ferrari et al., 2017). Furthermore, the Elongator acetyltransferase complex subunit 2 (*ELP2*) gene, a component of the Elongator complex which regulates the activity of RNA polymerase II, was identified as susceptible gene in patients with FTD and FTD-ALS (Dong et al., 2015; Taskesen et al., 2018). Additionally, Mishra et al. (2017) reported an association of *APOE* and the Translocase of outer mitochondrial membrane 40 (*TOMM40*) genes with bvFTD, and the Rho GTPase activating protein 35 (*ARHGAP35*) and the Serpin family A member 1 (*SERPINA1*) genes with progressive non-fluent aphasia. Further, they found the ε2 and ε4 alleles of *APOE* harboring protective and risk increasing effects, respectively, in FTD clinical subtypes (Mishra et al., 2017). *TOMM40* provided insight into a metabolic mitochondrial basis for the etiology of FTD (Roses et al., 2016); instead, the novel associations of *ARHGAP35* and *SERPINA1* with PNFA revealed a potential role of the stress-signaling pathway in FTD pathophysiology (Mishra et al., 2017). In 2015, the RFNG O-fucosylpeptide 3-beta-N-acetylglucosaminyltransferase (RFNG) and the Apoptosis-associated tyrosine kinase (AATK) genes, involved in neuronal genesis and differentiation and axon outgrowth, were recognized as genetic risk factors in an Italian FTD cohort (Ferrari et al., 2015). A GWAS on AD, FTD, and PSP evidenced the ATP binding cassette subfamily A member 7 (*ABCA7*), the Dysferlin (*DYSF*), and the PAX interacting protein 1 (*PAXIP1*) as susceptibility genes (Chen et al., 2015), known to be implicated in lipid metabolism, immune processes, mitochondrial abnormalities, and genome stability, respectively (Muñoz and Rouse, 2009; Vincent et al., 2016; Aikawa et al., 2018).

Although GWAS has played an important role in the discovery of risk variants for a specific trait, the identified loci are able to explain only a modest fraction of the predicted genetic variance. Technological limits, including small sample size, allelic heterogeneity and small effect sizes of these genetic variants, in addition to conceptual limitations, once again linked to the inability of common variants in explaining all the still remaining forms without an identified genetic factor, have again influenced the transition to a new approach: from common variants with small effect sizes to rare variants with a higher penetrance.

## Next-Generation Sequencing and the “Rare-Variants Theory”

A further significant contribution toward the knowledge of genetic FTD background came from the “rare-variants theory”: rare variants widespread in the genome could represent the missing genetic components for complex diseases. Interestingly, these variants can have determining effects on clinical phenotype, in terms of severity and earlier onset (Xu et al., 2018).

In this scenario, novel methodological issues have raised, due to the unavailability of suitable technologies to unravel the huge number of rare variants throughout the genome. The development of NGS has revolutionized the genetic research, allowing: the analysis of entire genomes (Whole Genome Sequencing, WGS); specific loci or selected candidate genes Targeted Sequencing (TS), or sequencing of exons of all coding genes (Whole Exome Sequencing, WES) (Pottier et al., 2015; Williams et al., 2016; Bonvicini et al., 2019). Thus, in this technological Era, a new opportunity is offered: the genetic analysis is no longer limited to the sequencing of the whole coding sequence of genes known for their implication in a disease, but it is also extended to the parallel analysis of groups of genes acting together in disease-relevant pathways (Boyle et al., 2017).

Thanks to NGS, the “rare variants hypothesis” has been explored also in FTD: discoveries achieved in this field in the last years are reported in Table 2.

**TABLE 2:** NGS mediated identification of rare variants associated with FTD.

Mutated gene	Main results	Approaches	References
*PSEN1, MAPT, APP*	- Identification of known and novel variants in *PSEN1*, *MAPT*, and *APP*	WES	Xu et al.,2018
	- FTD mutation carriers: low age of onset; more rapid progression		
*SNCA*	- Identification of a rare variant in *SNCA* in a bvFTD patient	NGS	Breza et al.,2018
	- Pleiotropic effect of *SNCA*		
	*-* Alterations of mitochondrial processes in FTD		
*VCP*	- Identification of known and novel variants in *VCP* both in family members and unrelated-cases	WES	Abrahao et al., 2016;
	- Pleiotropic effect of *VCP*→ heterogeneous clinical features		Saracino et al., 2018;
	- FTD associated with alterations of ubiquitin system, vesicle transport, proteostasis, neural vitality, and stress response		Wong et al.,2018
*SORT1*	- Identification of rare known and novel variants in *SORT1* in Belgian, Spanish, Italian, and Portuguese cohorts	NGS	Philtjens et al.,2018
	- *SORT1* further confirmed as genetic risk factor for FTD		
	- Defects in protein transport and cellular transduction		
*GRN, CSF1R*	- Identification of a *GRN* known pathogenic variant in a bvFTD case	NGS	Kim et al.,2018
*AARS2*	- Identification of novel variants in two dementia-related genes as *CSF1R* and *AARS2* in bvFTD patients		
	- Immunity response, inflammatory processes and mitochondrial function involvement in FTD etiology confirmed		
*TREM2, GRN*	- Identification of a rare variant in *TREM2*	TS	Ng et al.,2018
	- Identification of two novel nonsense *GRN* mutations		
	- *TREM2* and *GRN* further confirmed as FTD risk genes		
	- Immune pathways and inflammatory responses are altered in FTD		
*TYROBP*	- Identification of a rare variant in *TYROBP*	NGS	Giannoccaro et al.,2017
	- Immune pathway and inflammatory response involvement in FTD etiology confirmed		
*TBKBP1*	- Identification of novel deletions and missense mutations	TS	van der Zee et al.,2017
	- Immune response involvement in FTD etiology confirmed		
*SQSTM1*	- Identification of rare variants in an extended cohort of FTD patients	WES	van der Zee et al.,2014
	- Autophagy-lysosomal alterations in FTD		
*CCNF*	- NGS allowed to reveal, in a locus previously identified, a missense mutation in *CCNF*	NGS, linkage	Williams et al.,2016
	- Combined technologies for a better understanding of diseases	analysis	
	- Impairment of protein homeostasis mediated by *CCNF*		

Interestingly, a rare variant in the Alpha-synuclein (*SNCA*) gene, cause of autosomal dominant PD, was observed in a bvFTD patient, suggesting alterations in mitochondrial processes also in FTD (Mullin and Schapira, 2013; Breza et al., 2018). A WES study was conducted to perform a genetic exploration in patients with early onset forms of dementia, including FTD. Specifically, Xu et al. (2018) focused on 89 dementia-related causing and susceptible genes, identifying known pathogenic mutations in *PSEN1* (Presenilin 1) and *MAPT*, and one novel pathogenic variant in the Amyloid beta precursor protein (*APP*) gene. The authors also revealed that all the identified mutations caused dementia with an earlier age of onset and a more rapid disease progression (Xu et al., 2018).

Recently, a group of FTD subjects was screened for different known FTD genes through a WES approach: this study identified two novel and one already known *VCP* mutations in three patients with a clinical diagnosis of FTD (Wong et al., 2018). In addition, Saracino et al. (2018) analyzed *VCP* in an FTD cohort, observing seven mutations in unrelated families, including three novel mutations segregating with dementia. Interestingly, a novel rare missense variant in *VCP* was also described in a FTD subject, member of a family presenting an unusual intra-familiar association of a specific myopathy with ALS and FTD (Abrahao et al., 2016). In all these cases, NGS has permitted to reveal interesting mutations in *VCP*, implicated in ubiquitin pathways, vesicle transport, proteostasis, neural vitality and stress response (Meyer and Weihl, 2014; Rainero et al., 2017).

By gene target re-sequencing, rare variants within the Sortilin 1 (*SORT1*) gene were identified in a Belgian FTD cohort. A subsequent study of cohorts sampled in Spain, Italy and Portugal revealed additional non-synonymous variants in European patients. Specifically, *SORT1* is a known FTD risk factor: the encoded protein is a neuronal receptor involved in intracellular protein transport and cellular signal transduction (Philtjens et al., 2018).

In sporadic FTD patients without a recognized genetic cause in the well-known FTD related genes (*MAPT*, *GRN*, and *C9orf72*), novel variants were identified in two dementia-related genes, the Colony stimulating factor 1 receptor (*CSF1R*) and the Mitochondrial alanyl-tRNA synthetase 2 (*AARS2*), suggesting new genes to be considered for a genetic FTD diagnosis. *CSF1R*, which shows important role in innate immunity and inflammatory processes, and *AARS2*, involved in mitochondrial functions, highlight alterations of these processes in the FTD etiology (Kim et al., 2018).

Recently, a TS of 12 FTD-associated genes was performed: this study revealed a rare variant in the Triggering receptor expressed on myeloid cells 2 (*TREM2*) and two nonsense *GRN* mutations (Ng et al., 2018).

In Giannoccaro et al. (2017), a panel of dementia-associated genes was explored in an Italian group of ALS/FTD pedigrees by using a TS approach: genetic variants in additional ALS and dementia-related genes were found in four pedigrees, including a rare variant in the Tyrosine kinase binding protein (*TYROBP*) gene. The *TYROBP* protein, which interacts with several other proteins like *TREM2*, is specifically involved in immune pathway and inflammatory response (Giannoccaro et al., 2017).

In addition, the TBK1 binding protein 1 (*TBKBP1*) was screened in a wide cohort of FTD, ALS, FTD-ALS subjects through a TS approach, identifying deletions and missense mutations in this gene involved in immune response (van der Zee et al., 2017).

In van der Zee et al. (2014), rare variants in the Sequestosome 1 (*SQSTM1*) gene were identified in a cohort of FTD patients, suggesting a role of this gene in the etiology of disease.

Next-Generation Sequencing coupled with conventional approaches is considered the cutting-edge approach for a better understanding of the genetic underpinnings of complex diseases: studies employing NGS have identified rare variants within regions previously prioritized by GWAS, along with novel variants in previously unidentified genes (Williams et al., 2016; Patel et al., 2017). As for GWAS, the linkage analysis has again emerged as an extremely powerful method for the identification of variants implicated in disease in conjunction with WGS filtering approaches (Ott et al., 2015). As regards, in Williams et al. (2016) a genome-wide linkage analysis identified a novel disease locus on chromosome 16p13.3 in a large ALS/FTD cohort. NGS allowed to reveal at this locus a novel missense mutation in Cyclin F (*CCNF*) gene, in which specific mutations have been subsequently described in FTD-ALS subjects (Lee et al., 2018), pointing toward an impairment of protein homeostasis in this complex disorder (Williams et al., 2016; Pan et al., 2017).

## Concluding Remarks

Overall, this mini-review points up that GWAS and NGS, based on the analysis of different variants with moderate-to-strong effect, have concurrently revealed the implication of common molecular pathways in FTD. In particular, these approaches revealed genetic alterations in genes acting together in molecular pathways involved in neuronal-viability and survival, vesicle trafficking, immune and inflammatory response, and energy metabolism. Noteworthy, it has been suggested that defects of all these primary processes could be interrelated at different levels, leading to the degeneration of the whole system and, thus, causing the disease (Ramanan and Saykin, 2013). In particular, multiple studies consolidate the view that immune and endo/lysosomal processes are key players in the pathobiology of these disorders. In future studies, the combination of different molecular approaches also at protein and metabolic levels will definitely help in further clarifying the role of these pathways in FTD pathogenesis and their possible interconnection. In this way, we will foster our potential to move toward effective prevention and therapy for this incurable neurodegenerative disease.

## Author Contributions

MC, LB, CB, and RG gave their substantial contribution to conception and design of the manuscript and drafting the manuscript, revising it critically for important intellectual content. All authors have approved the manuscript in its present form for publication. All authors agreed to be accountable for all aspects of the work in ensuring that questions related to the accuracy or integrity of any part of the work are appropriately investigated and resolved.

## Conflict of Interest Statement

The authors declare that the research was conducted in the absence of any commercial or financial relationships that could be construed as a potential conflict of interest.

## Footnotes

1 http://www.molgen.ua.ac.be/ADMutations/

2 https://www.alzforum.org
